# Balancing health care needs in a changing context: nursing highlights from the 2016 European Oncology Nursing Society Congress (EONS10), 17–18 October 2016, Dublin, Ireland

**DOI:** 10.3332/ecancer.2017.710

**Published:** 2017-01-05

**Authors:** Danuta Lichosik, Rosario Caruso

**Affiliations:** 1 IEOEDUCATION, School of Robotic Surgery, European Institute of Oncology, 20141 Milan, Italy; 2 Health Professions Research and Development Unit, IRCCS Policlinico San Donato, 20097 San Donato Milanese, Italy

**Keywords:** clear cell adenocarcinoma of the cervix, radical trachelectomy, chemotherapy

## Abstract

European cancer nurses have to face many challenges as a result of the rapidly changing economic and political context in which balancing health care needs has become strategic for healthcare delivery. Currently, cancer nurses must overcome many obstacles arising from clinical, organisational, and educational issues. Within this scenario, the European Oncology Nursing Society (EONS) shaped its tenth congress programme to boost discussion and reflections, to share experiences and research, and to see how cancer nurses try to anticipate and embrace changes. The aim of this was to promote innovative solutions and to address the many issues involved with cancer care.

EONS10 was held on 17–18 October in Dublin, Ireland. The congress was attended by more than 500 delegates. The programme covered the following themes: caring for families and carers, inequalities and access to cancer care, caring for patients with haematological cancers, palliative care, communication and information exchange, community cancer care (i.e. parallel sessions), roles and responsibility for advanced nursing practice, International Psycho-Oncology Society (IPOS)-Academy workshops (i.e. workshops), cancer survivorship, clinical leadership and new roles, oncology nursing research, symptom experiences and management, palliative care (i.e. proffered papers), poster presentations, and satellite symposia. The aim of this paper is to highlight and discuss the contents of the EONS10 congress.

## Introduction

The context in which cancer nurses work in Europe is rapidly changing, considering the challenges brought about by demographic changes (i.e. ageing population), treatment innovations and complexity, the need to deliver care for cancer patients, for their families and survivors, issues related to the nursing workforce, competencies [1], and the political climate. EONS structured its tenth congress (EONS10) programme in continuity from the previous year’s congress [2] and in synergy with other European Cancer Congresses [3, 4]. EONS10 was held on 17–18 October in Dublin (Ireland), and it was attended by more than 500 delegates. This provided a unique opportunity to share ideas, present research findings, and to debate the main issues related to cancer nursing practice and innovations.

Thus, cancer nurses and other key members of multi-professional teams discussed here the care needs of patients, their families, and the challenges nurses have to face in order to deliver care in a fast-changing world. This is particularly significant in a field where innovation develops quickly in every nuance of clinical practice such as cancer care. They addressed the weaknesses between different approaches and profiles. EONS10 was also a unique occasion to thoroughly understand the importance of roles within the nursing workforce given the increasing number of cancer patients and therapeutic options available. This all involves providing education and supportive care to patients and their families by them.

Considering the varied and important issues discussed at EONS10, a conference report could be useful to summarise the main topics presented. For this reason, the aim of this paper is to highlight and discuss the contents of the EONS10 congress.

## EONS10 congress

The EONS10 congress opened with welcome speeches from the EONS president (Professor Daniel Kelly), the Irish Association for Nurses in Oncology (IANO) president (Pauline Kehoe), the chair of the EONS10 scientific committee (Professor Mary Wells), and the minister for health of Ireland (Mr Simon Harris). Then, Professor Meinir Krishnasamy (University of Melbourne, Australia) gave a lecture on the congress topic ‘Balancing health care needs in a changing context’, highlighting (a) how the global healthcare industry is in crisis because of the increase of cancer epidemiology worldwide, (b) the challenges brought about by personalised cancer therapies and technology, (c) the role of prevention (e.g. a third of new cancer cases can be prevented through modification of lifestyle behaviours and access to screening), (d) the importance of enhancing cancer patient outcomes, (e) and key reflections to redress the balance of care needs in a changing global context.

This paper is structured in five main sections to highlight the contents of EONS10 [5] which are:

- parallel sessions
- proffered papers
- workshops
- poster presentations
- satellite symposia

### Parallel sessions

A session entitled ‘Caring for families and carers’ was chaired by Professor Daniel Kelly (UK, EONS President). The first presentation of this session entitled ‘Supporting parents with cancer who have young children’, was presented by Cherith Semple (UK), and it aimed to describe how nurses can support the needs of parents diagnosed with cancer who have young children. The second presentation was presented by Sylvie Lambert (Canada), and it aimed to show the findings of a longitudinal study on caregivers’ well-being over the first five years after diagnosis. Naomi Fitzgibbon (Ireland) gave the third presentation which aimed to discuss traditional and non-traditional technologies to support cancer patients and their families.

The session titled ‘Inequalities and access to cancer care’ was chaired by Andreas Charalambous (Cyprus) and Aoife McNamara (Ireland). Edel McGinnity (Ireland) highlighted how much the current structure of health services discriminates against patients with the highest health need, showing the specific challenges of delivering care in areas of deprivation, and how to improve access and outcomes in these areas. Kathi Apostolidis (Greece) presented a communication aimed at raising awareness about inequalities in cancer care across Europe, suggesting some tips, and a political agenda to tackle the issue of health inequalities. Then Nick Hulbert-Williams (UK) presented a lecture on cancer experiences in demographic minority groups. He showed how the variation in sociodemographic characteristics (i.e. age, gender, ethnicity, minority groups) can affect a range of healthcare experiences. His presentation also pointed out the experiences of cancer in people with an intellectual disability and the inequalities existing between sexual orientation groups.

The session entitled ‘Caring for patients with haematological cancers’ was chaired by Barry Quinn (The Netherlands) and Patrick Crombez (Belgium), and it was in conjunction with the European Group for Blood and Marrow Transplantation (EBMT). The first speaker, Melanie Charalambous (Cyprus), described the pathophysiology of oral mucositis, highlighting its pharmacological and non- pharmacological management. The second speaker was Barry Quinn (UK) who pointed out the important role of patients’ self-assessment and personalised information aimed at providing better choices and health for patients. The third speaker was Susan Schneider (USA) and she spoke about nutritional support in high dose chemotherapy, highlighting how there is a lack of research on that topic.

Another interesting session was entitled ‘Communication and information exchange’, and it was chaired by Paul Trevatt (UK) and co-chaired by Gabriella Pravettoni (Italy). The first speaker was Lena Sharp (Sweden) with a presentation entitled ‘Nursing handover sessions’, highlighting how nursing handover could be a potential threat to patient safety, and how a lack of research still exists with the aim of comparing the effectiveness of different models of handover. She also described the experience of Karolinska University Hospital (Sweden) in implementing a person-centred bedside nursing handover. The second speaker was Paul D’Alton (Ireland) and his presentation described the ways in which nurses can respond more effectively in different interpersonal situations. Then the third speaker proposed some reflections on sharing knowledge about death and dying at different levels (i.e. public, Health Care Systems, patients, and relatives).

The session entitled ‘Community cancer care’ was chaired by Eileen O’Donovan (Ireland) and discussed by Theresa Wiseman (UK). The first speaker was Janice Richmond (Ireland) who proposed an integrated approach to face cancer challenges, establishing a community oncology nursing programme to improve cancer care. The second speaker was Eila Watson (UK) with a presentation which described the role of primary care for cancer survivors. The third speaker, Natasha Campling (UK), described the implications of opioid medicines management at home for patients, nurses, and carers.

The session ‘Cancer survivorship’ chaired by Lena Sharp (Sweden) and discussed by Mary Wells (UK) highlighted (a) the role of physical exercise, (b) the alternative models of cancer survivors follow-up, (c) the role of self-efficacy in recovery following cancer treatment, and (d) the role of patients’ self-management. Then, the sessions ‘Alternative and complementary approaches’ chaired by Andreas Chralambous (Cyprus) and discussed by Iveta Nohovovà (Czech Republic) aimed (a) to describe the current research challenges in complementary and alternative therapies (CAM), (b) to describe the power of therapeutic touch for people living with a diagnosis of cancer, (c) to report some patients’ experiences of CAM.

The session ‘Psychological health and wellbeing in nurses’ was chaired by Erik van Muilekom (The Netherlands) and was discussed by Rosario Caruso (Italy). This session aimed (a) to describe what compassion fatigue is for nurses, (b) to describe European policy on how to face the challenges of stress in cancer nursing, and (c) to describe an experience of professional supervision to improve nurses’ coping. Another interesting session, entitled ‘Patient reported outcomes’, was chaired by Wendy Oldenmenger (The Netherlands) and was discussed by Patrich Jahn (Germany). It aimed (a) to describe the main patient-reported outcomes, (b) to understand psychosocial difficulties, and (c) to describe an experience of patient reported outcomes at a population level in Ireland.

### Proffered papers

The EONS10 congress was very rich in proffered papers which were selected by the scientific committee from among many abstracts. During the congress, delegates had the opportunity to choose which proffered papers sessions they would attend after considering the different proposed topics. There were seven sessions related to proffered papers, and the presentations were grouped according to the following topics: (a) Long term consequences and cancer survivorship; (b) Clinical leadership and new roles; (c) Oncology nursing research; (d) Symptom experiences and management; (e) Palliative care; (f) New developments and roles; (g) Psychosocial care.

### Workshops

EONS10 was also a great occasion to attend interesting workshops dealing with some important challenges related to cancer care delivery. The challenges addressed by those workshops were concerning (a) the roles and responsibilities of advanced nursing practitioners, (b) the management of psychological distress for cancer patients, (c) ways to help parents with advanced cancer to talk with their children, (d) and a discussion on the myths and challenges related to living and dying for cancer patients.

### Poster presentations

More than 200 posters were displayed covering the most challenging areas of cancer nursing practice such as advanced nursing roles, end of life care, impact of cancer on patients and families, new developments, supportive and palliative care, survivorship and rehabilitation, symptom management and transitions in care. Many posters were presented by authors coming from different countries in discussion sessions, characterised by interesting debates where delegates shared their experiences, also strengthening their networks. Poster presentations have been shown to be a high quality method for knowledge exchange.

### Satellite symposium

Delegates also had the opportunity to participate in many interesting satellite symposia dedicated to some innovative cancer treatments. Among these symposia, ecancer organised a session entitled ‘New optimism for advanced breast cancer patients failing antioestrogens’, available on the ecancer website at the following address: http://ecancer.org/education/module/266-new-optimism-for-advanced-breast-cancer-patients-failing-antiestrogens.php.

## Conclusion

EONS10 was a high quality conference aimed at discussing how nurses could help to balance healthcare needs in a changing context. EONS10 was a unique occasion to thoroughly understand how the nursing workforce has an important role in meeting the increasing needs of an increasing amount of cancer patients. Moreover, the congress offered a fantastic opportunity to the delegates to strengthen their networks and to be involved with the challenges brought about by the current epidemiological and economic scenario.
